# Clinical Efficacy, Mechanisms, and Safety of Acupuncture and Moxibustion

**DOI:** 10.1155/2014/356258

**Published:** 2014-02-04

**Authors:** Jaung-Geng Lin, Yi-Hung Chen, Xin-Yan Gao, Lixing Lao, Hyejung Lee, Gerhard Litscher

**Affiliations:** ^1^School of Chinese Medicine, China Medical University, Taichung 40402, Taiwan; ^2^Graduate Institute of Acupuncture Science, China Medical University, Taichung 40402, Taiwan; ^3^Department of Physiology, Institute of Acupuncture and Moxibustion, China Academy of Chinese Medical Sciences, Beijing 100700, China; ^4^School of Chinese Medicine, The University of Hong Kong, 10 Sassoon Road, Pokfulam, Hong Kong; ^5^Acupuncture and Meridian Science Research Center, College of Korean Medicine, Kyung Hee University, Seoul 130-701, Republic of Korea; ^6^Stronach Research Unit for Complementary and Integrative Laser Medicine, Research Unit of Biomedical Engineering in Anesthesia and Intensive Care Medicine and TCM Research Center, Medical University of Graz, 8036 Graz, Austria

Acupuncture has recently increased in popularity and is widely used all over the world. It is described as one of the “complementary and alternative medicine/therapies,” showing promising efficacy in the treatment of many conditions and resulting in fewer adverse effects compared with some conventional medical treatments. Many studies in animals and humans have demonstrated that acupuncture results in multiple biological responses. Although the endorphin hypothesis is well established, the mechanisms underlying acupuncture treatments have not been extensively studied. Basic and clinical acupuncture studies are important and timely. Although acupuncture has a relatively sound safety profile, adverse effects after acupuncture have been reported. More information is needed on safe needling depths. Moxibustion is another traditional Chinese medical intervention that involves the burning of moxa above the body surface of the acupuncture points. The clinical efficacy and mechanism of moxibustion have not been extensively studied. Moreover, safety issues related to moxibustion safety need to be investigated, including concerns of potential tissue damage and adverse physical reactions.

In an attempt to summarize the current knowledge on clinical efficacy, mechanisms, and safety of acupuncture and moxibustion, this special issue contains 38 interesting publications: 14 describe the clinical effects of acupuncture; 15 describe the mechanisms of acupuncture; 3 concern the safety of acupuncture; 2 concern the clinical effects of acupressure; 2 detail clinical effects of moxibustion; and 2 describe the mechanisms of moxibustion. The investigations cover *in vitro* investigations, preclinical experiments, and studies in healthy volunteers and patients, as well as basic and clinical research.

The above-mentioned papers investigate the role of acupuncture in the following therapeutic areas: (1) pain, (2) the respiratory system, (3) microcirculation, (4) neurodegeneration, (5) the endocrine system, (6) gastric motility, (7) itch, (8) seizures, (9) neuroprotection, (10) opioid receptors, (11) diabetes mellitus, (12) public health issues of acupuncture, (13) the effects of acupuncture on infants, (14) adverse effects of acupuncture, (15) safe needling depth, (16) psychology during acupuncture, (17) acupressure and dysmenorrhea, (18) acupressure and sleep disturbance, (19) moxibustion and hyperlipidemia, and (20) a discussion of the mechanisms and safety of moxibustion.

Nowadays, modern technologies are being used to explore the effects of acupuncture and moxibustion. The results of these investigations enhance our knowledge of how acupuncture and moxibustion work and promote better insight into how best to use these tools.

